# Dataset of bimanual human-to-human object handovers

**DOI:** 10.1016/j.dib.2023.109277

**Published:** 2023-05-27

**Authors:** Alap Kshirsagar, Raphael Fortuna, Zhiming Xie, Guy Hoffman

**Affiliations:** a Sibley School of Mechanical and Aerospace Engineering, Cornell University, Ithaca, NY, USA; b Electrical and Computer Engineering, Cornell University, Ithaca, NY, USA

**Keywords:** Human-robot interaction, Human motion, Robotics, Collaborative manipulation

## Abstract

We present a multi-sensor dataset of bimanual human-to-human object handovers. The dataset consists of 240 recordings obtained from 12 pairs of participants performing bimanual object handovers with 10 objects, and 120 recordings obtained from the same 12 pairs of participants performing unimanual handovers with 5 of those objects. Each recording includes the giver and receiver's 13 upper-body bone position and orientation trajectories, position trajectories for the 27 markers placed on their upper bodies, object position and orientation trajectories, and two RGB-D data streams. The motion trajectories are recorded at 120Hz and the RGB-D streams are recorded at 30Hz. The recordings are annotated with the three handover phases: reach, transfer, and retreat. The dataset also includes four anthropometric measurements of the participants: height, waistline height, arm span, and weight. Our dataset could help investigations of the bimanual reaching motions and grasps utilized by humans while performing handovers. Also, it can be used to train robots to perform bimanual object handovers with humans.

**Specifications Table**

**Table:** 

Subject	Engineering
Specific subject area	Human-Robot Interaction
Type of data	Tables and Videos
How the data were acquired	Motion Capture (Optitrack Flex 13); RGB-D camera (Microsoft Kinect v2.0); Measuring Tape; Weighing Scale
Data format	Raw and processed .csv, .mp4, .mat
Description of data collection (400 characters)	Pairs of participants performed the object handovers standing face-to-face, and the giver held the object before the start of the handover. A motion capture system and two RGB-D sensors were used to record these object handovers between each pair of participants.
Data source location	Institution: Cornell University City/Town/Region: Ithaca Country: United States of America Latitude and Longitude: 42.4439° N, 76.4820° W
Data accessibility	Repository name: Zenodo Data identification number: 10.5281/zenodo.7767535 Direct URL to data: https://zenodo.org/record/7767535#.ZB2-43bMLIU

## Value of the Data

• This dataset is useful for studying various features of bimanual handovers between two persons, such as handover location, object orientation, grasp configuration, and reaching velocity profile.
• This dataset can be used to build and evaluate models of human motion in bimanual handovers, a task that people frequently perform in their daily lives.
• This dataset can be used to train a robot to perform the tasks of human-to-robot and robot-to-human handovers which are essential for human-robot collaboration.
• This dataset can be used to compare bimanual handovers and unimanual handovers, which can inspire new motion models and robot controllers for object handovers.
• This dataset can benefit researchers studying human-robot interaction, robotic manipulation, and human motor skills.

## 1 Objective

There are numerous applications where bimanual handovers, i.e. handing over objects with two handed grasps, are useful or even necessary. Bimanual handovers are necessary when handing over large rigid objects, deformable objects, spherical objects, and delicate objects. Also, in some cultures, it is a rule of etiquette to hand over objects with two hands. The existing datasets of human-to-human handovers [1], [2], [3], [4] only consider handovers of objects with single-handed grasps i.e. uni-manual handovers. To the best of our knowledge, there is no public dataset of human-to-human handovers of objects requiring bimanual grasps, even though such handovers are equally important and even more challenging. Our dataset is aimed at addressing this gap.

### 1.1 Data Description

**Fig. 1:**
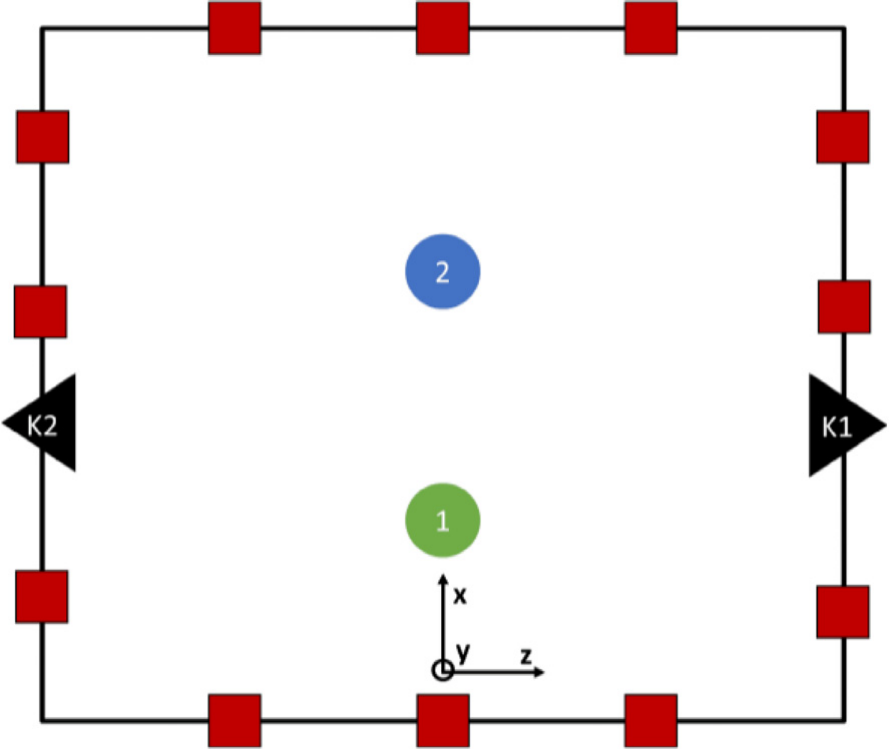
Schematic of the data collection setup of the dataset. We used an OptiTrack motion tracking system with 12 cameras (red squares), and two Kinect v2 sensors (black triangles). The green and blue circles represent the positions of the participants. The reference frame in the Fig. shows the reference frame of the motion tracking system whose origin was on the floor and behind the participant shown with the green circle. The participants were instructed to stand along the x-axis of this reference frame, at an interpersonal distance that is comfortable to them.

**Fig. 2:**
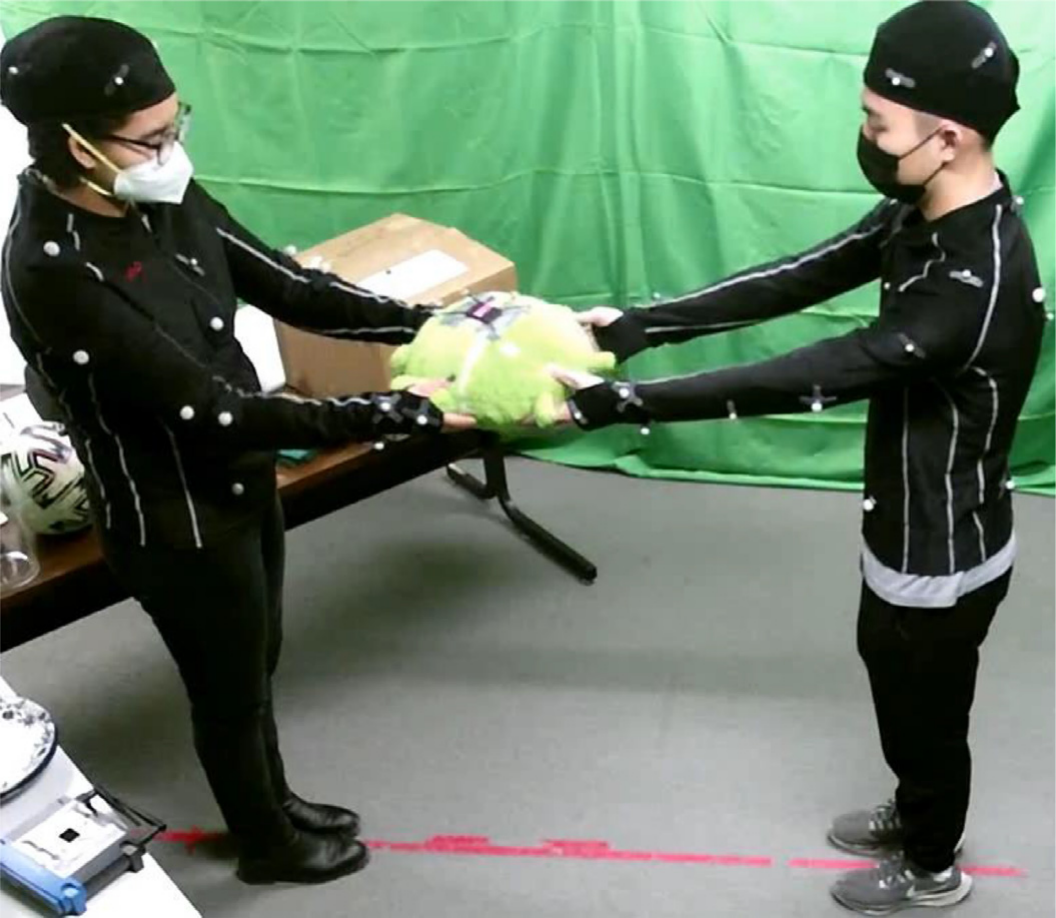
Example of a bimanual handover between two participants. The participants are wearing motion capture suits with 27 reflective markers per suit. A set of 6 reflective markers is attached to the object.

**Fig. 3:**
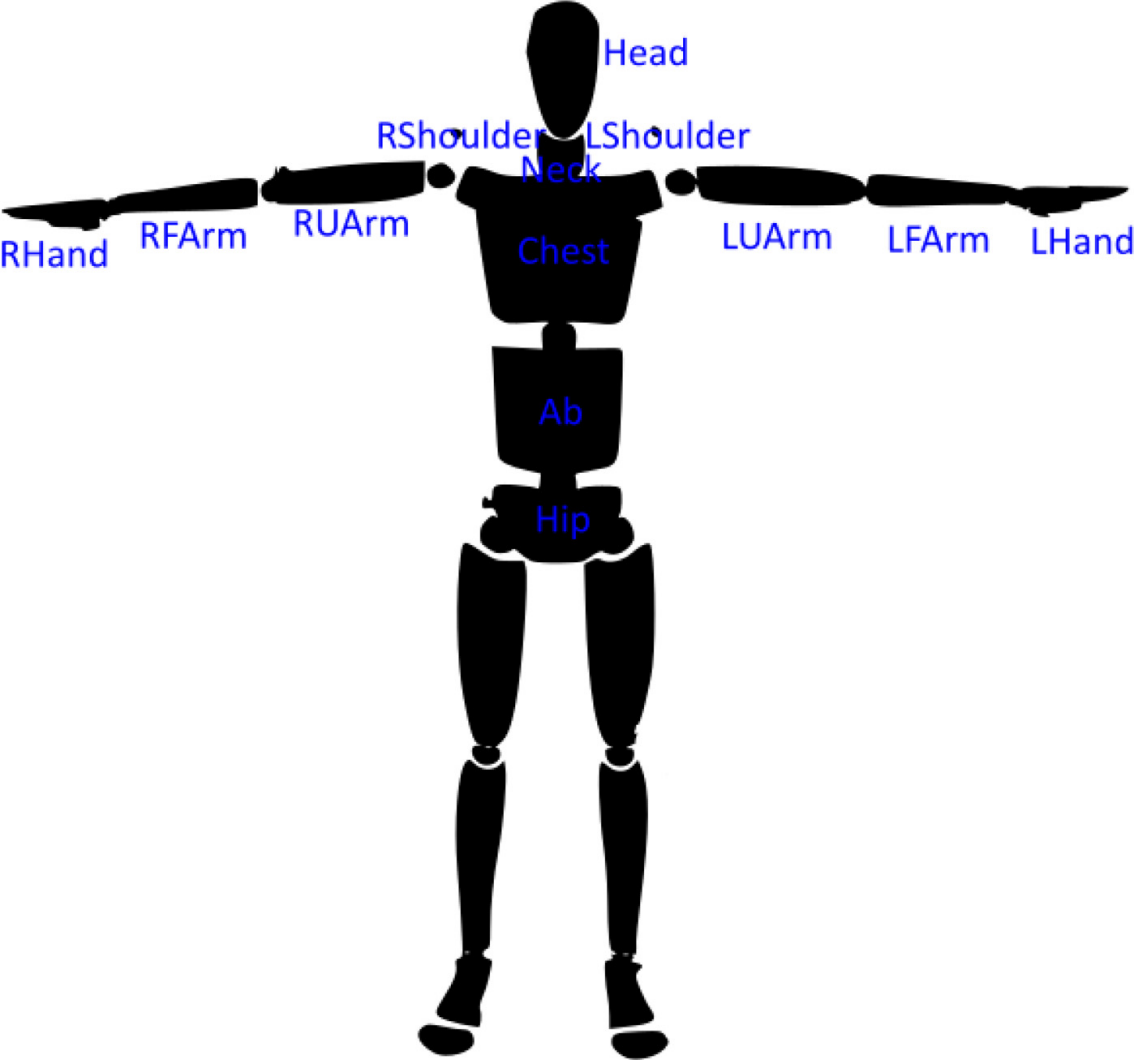
Locations of the 13 bones in the "Conventional Upper Body'' skeleton.

**Fig. 4:**
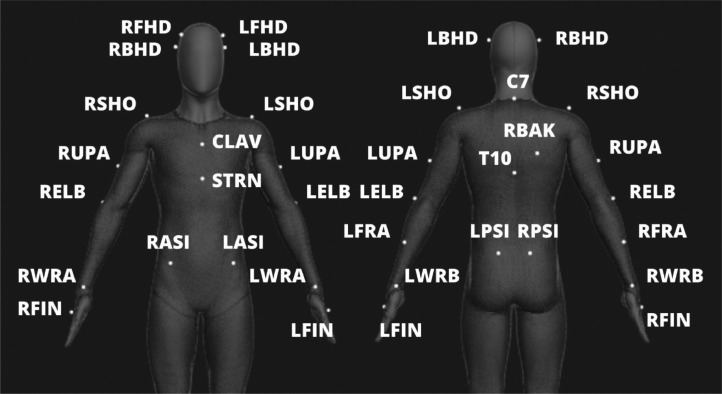
Locations of the 27 bone markers in the ``Conventional Upper Body'' skeleton.

This dataset contains 240 recordings collected from 24 volunteers performing bimanual handovers, i.e. handovers with two-handed grasps (Fig. 1, Fig. 2), with 10 objects (Fig. 5 and Table 3). We also include 120 recordings from the same volunteers performing unimanual handovers, i.e. handovers with single-handed grasps, with 5 of those objects (object numbers 1 to 5 in Fig. 5). Each recording consists of:

1. The Cartesian position trajectories and the Quaternion orientation trajectories of 13 upper-body bones for each participant, the Cartesian position trajectories of 27 upper-body bone markers for each participant, and the Cartesian position trajectory and the Quaternion orientation trajectory of the object. Fig. 3 shows the locations of 13 upper-body bones and Fig. 4 shows the locations of the 27 upper-body bone markers. All the trajectories are recorded with an OptiTrack Flex 13 Motion Capture System and are in the reference frame of the motion tracking system. The trajectories are annotated with the three handover phases of the giver and receiver: reach, transfer, and retreat. Each of these files is a 355-dimensional representation of a handover.

**Fig. 5:**
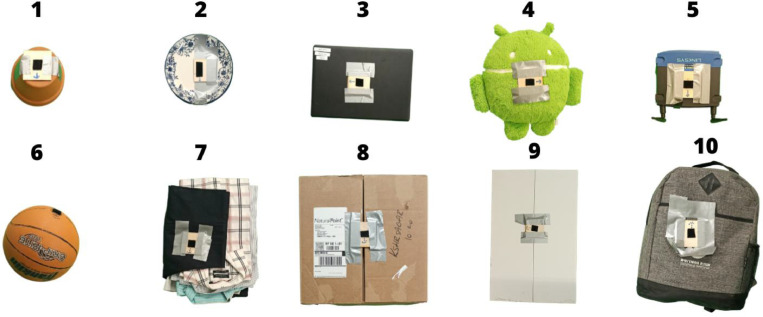
Objects used in the dataset. The object names and properties are listed in Table 3. Each object is fitted with a loop part of the hook-and-loop fastener to facilitate easy attachment and removal of the marker-set.
2. The Quaternion orientations of 13 upper-body bones for each participant with respect to their parental segment, generated by the OptiTrack Motive software, which roughly represent the joint angles. Each of these files is a 108-dimensional representation of a handover.
3. RGB videos and Depth data recorded with two Microsoft Kinect v2 sensors at 30fps. Each video frame has a resolution of 1920 × 1080 and each depth frame has a resolution of 512 × 424.

The dataset also includes the height, weight, arm span, and waistline height of the participants.

The dataset contains one subfolder for each participant pair, named “P[ab]_P[cd]” where “ab” and “cd” are the two-digit participant numbers*.* Each of these subfolders contains four subfolders:

1. Kinect_1: This subfolder contains the data obtained from the first Kinect v2 sensor. There are three types of files inside this folder: “Pab_Pcd_HandoverType_Object.mp4”, “Pab_Pcd_HandoverType_Object_Depth.mat”, and “Pab_Pcd_HandoverType_Object_Depth_Timestamps.csv”. In all three filenames, “Pab” corresponds to the participant number of the giver, “Pcd” corresponds to the participant number of the receiver, “HandoverType” is “double” for bimanual handovers and “single” for right-handed handovers, and “Object” is the name of the object being handed over. There are 10 objects: bag, basketball, cardboard, clothes, dishes, laptop, large cardboard box (lbox), plush toy, pot, and router.

The “.mp4” file is the RGB video recording of a handover with a resolution of 1920 × 1080 at 30fps.

The “.mat” file is the depth data of a handover with a resolution of 512 × 424.

The timestamp of each depth frame with reference to the first video frame is provided in the “.csv” file.

2. Kinect_2: This subfolder contains the data obtained from the second Kinect v2 sensor. The file types inside this folder are similar to those in the Kinect_1 folder.
3. OptiTrack_Global_Frame: This subfolder contains the data obtained from the OptiTrack Flex 13 Motion Capture System. Due to the occlusions of markers, a total of 0.65% values are missing in this data. Each file inside this folder is named in the format “Pab_Pcd_HandoverType_Object.csv”, similar to the Kinect files. For example, “P07_P08_double_bag.csv” corresponds to the bimanual handover of the “bag” from “P07” to “P08”. Each file contains the trajectories of 13 upper-body bones and 27 upper-body bone markers for both the participants and the trajectory of the object. All trajectories are in the reference frame of the motion tracking system and are recorded at 120fps. The trajectories are annotated with the handover phases (reach/transfer/retreat) of both the participants and the index and timestamp of each dataframe. Thus, each of these files is a 355-dimensional representation of a handover. The column headers in these files are described below:

“Index” - Dataframe index, starts with 0

“Giver” - Handover phase of the giver, reach/transfer/retreat

“Receiver” - Handover phase of the receiver, reach/transfer/retreat

“Time” - Dataframe timestamp in seconds.milliseconds

“Structure:Pab:Name:TrajectoryType:Axis” - Trajectory of a bone or a bone marker of the participant “Pab”. “Structure” is either “Bone” or “Bone Marker”. For bones, the “Name” is one of the 13 bone names listed in Table 1 and shown in Fig. 3. For bone markers, the “Name” is one of the 27 marker names listed in Table 2 and shown in Fig. 4. “TrajectoryType” is either “Position” or “Orientation”. The positions are in meters (“Axis” = X or Y or Z) and the orientations are in quaternions (“Axis” = X or Y or Z or W).

**Table 1:** Names and acronyms of the 13 bones in the “Conventional Upper Body” skeleton.

Bone Name	Acronym
Head	Head
Right Shoulder	RShoulder
Left Shoulder	LShoulder
Neck	Neck
Chest	Chest
Abdomen	Ab
Hip	Hip
Right Upper Arm	RUArm
Left Upper Arm	LUArm
Right Forearm	RFArm
Left Forearm	LFArm
Right Hand	RHand
Left Hand	LHand

**Table 2:** Names and acronyms of the 27 bone markers in the “Conventional Upper Body” skeleton.

Bone Marker Name	Acronym	Bone Marker Name	Acronym
Right Front Head	RFHD	Right Posterior Superior Iliac Spine	RPSI
Left Front Head	LFHD	Left Posterior Superior Iliac Spine	LPSI
Right Back Head	RBHD	Right Anterior Superior Iliac Spine	RASI
Left Back Head	LBHD	Left Anterior Superior Iliac Spine	LASI
Right Shoulder	RSHO	Right Wrist Bar (Thumb Side)	RWRA
Left Shoulder	LSHO	Left Wrist Bar (Thumb Side)	LWRA
Right Upper Arm	RUPA	Right Wrist Bar (Little Finger Side)	RWRB
Left Upper Arm	LUPA	Left Wrist Bar (Little Finger Side)	LWRB
Right Elbow	RELB	Clavicle	CLAV
Left Elbow	LELB	Sternum (Xiphoid Process)	STRN
Right Forearm	RFRA	7^th^ Cervical Vertebrae (Spinous Process)	C7
Left Forearm	LFRA	Right Back (Mid Scapula)	RBAK
Right Finger	RFIN	10^th^ Thoracic Vertebrae (Spinous Process)	T10
Left Finger	LFIN		

“Rigid Body:object:TrajectoryType:Axis” - Trajectory of the object being handed over. “TrajectoryType” is either “Position” or “Orientation”. The positions are in meters (“Axis” = X or Y or Z) and the orientations are in quaternions (“Axis” = X or Y or Z or W).

4. OptiTrack_Local_Frame: This subfolder contains the quaternion orientation trajectories of 13 upper-body bones for each participant with respect to their parental segment, generated by the OptiTrack Motive software, roughly representing the joint angles. Thus, each of these files is a 108-dimensional representation of a handover. Due to the occlusions of markers, a total of 0.59% values are missing in this data. The filename and column header format is the same as the files in “OptiTrack_Global_Frame” excluding the bone markers and the object.

The dataset also includes a “participant_anthropomorphic_measurements.csv” file which contains the height (in meters), weight (in kilograms), arm span (in meters), and waistline height (in meters) of the participants.

### 1.2 Experimental Design, Materials and Methods

We conducted a user study to create a multi-sensor dataset of bimanual human-to-human handovers [5]. This section describes the study protocol and data collection process.

• **Study Setup**

Our experiment setup, shown in Fig. 1, consisted of an OptiTrack motion tracking system with 12 Flex-13 cameras, and two Kinect v2 sensors. The area was surrounded by green screens to make it easier for other researchers to do post-processing on the video recordings in the future.

• **Motion Tracking System**

The OptiTrack cameras were connected to a desktop computer (CPU: Intel Core i5-7600 3.5GHz, GPU: 2GB AMD Radeon T R5 430 and 4 GB Intel HD Graphics 630, RAM: 8GB, running Windows 10 Pro) which recorded the skeleton tracking data through OptiTrack's Motive software. To track the participants' motion in bimanual handovers, we used the “Conventional Upper Body” marker-set consisting of 27 reflective markers placed at the positions shown in Fig. 4. Along with the 3D positions of these 27 markers, Motive also computes—from the marker positions—the 3D position and orientation of 13 upper-body bones shown in Fig. 3.

• **Kinect Sensor**

The first Kinect v2 sensor (K1 in Fig. 1) was connected to a desktop computer (CPU: Intel Core i7-6700 3.4GHz, GPU: 4GB NVIDIA Quadro P1000, RAM: 16GB, running Windows 10 Pro) which recorded the data streams from the Kinect sensor in the Microsoft Kinect Studio software. The second Kinect v2 sensor (K2 in Fig. 1) was connected to a laptop (CPU: Intel Core i7-7700HQ 2.8GHz, GPU: 4GB NVIDIA GeForce GTX 1050 and 8GB Intel HD Graphics 630, RAM: 16GB, running Windows 10 Home) which recorded the data streams from the Kinect sensor in the Microsoft Kinect Studio software. The Kinect v2 provides RGB-D streams in the following format: i) RGB - Resolution of 1920 × 1080 ii) D - Resolution of 512 × 424. Both streams are recorded at a frame rate of 30 fps. If the light is low, the sensor changes to 15 frames per second to allow in more light for better exposure.

• **Handover Objects**

We used 10 objects of different shapes, sizes, rigidity, and fragility, as shown in Table 3 and Fig. 5, in the study.

**Table 3:** Characteristics of objects used in the bimanual handovers dataset. D: Diameter, H: Height.

#	Name	Geometry	Size (mm)	Weight (kg)	Rigidity	Fragility
1	Flowerpot	Cylinder	153 D, 112 H	0.6	Hard	Fragile
2	Dishes	Cylinder	250 D, 80 H	1.61	Hard	Fragile
3	Laptop	Cuboid	342 × 240 × 25	1.36	Hard	Fragile
4	Plush Toy	Cylinder	380 D, 150 H	0.35	Deformable	Non-fragile
5	Wifi Router	Cuboid	200 × 173 × 45	0.38	Hard	Fragile
6	Basketball	Spherical	240 D	0.6	Hard	Non-fragile
7	Folded Clothes	Cuboid	420 × 300 × 70	1.04	Deformable	Non-fragile
8	Cardboard Box	Cuboid	400 × 390 × 280	1.16	Hard	Non-fragile
9	Cardboard Plank	Cuboid	560 × 355 × 15	0.21	Hard	Non-fragile
10	Backpack	Cuboid	460 × 395 × 35	1.3	Deformable	Non-fragile

• **Study Protocol**

After entering the room containing the recording setup, participants signed the consent form and wore the motion capture suits. The experimenter calibrated the skeletons in the Motive software for each participant. Then the participants performed both unimanual (right-handed) and bimanual handovers of 5 objects shown in Fig. 5 (top row) and only bimanual handovers of 5 objects shown in Fig. 5 (bottom row). They performed these handovers standing face-to-face. They were instructed to stand along the x-axis of the reference frame of the motion tracking system (see Fig. 1), at an interpersonal distance that is comfortable to them. Finally, the experimenter measured the height, weight, arm span, and waistline height of the participants.

• **Participants**

A total of 30 volunteers (15 pairs) participated in our study. Out of these, the first three pairs i.e. six participants were pilot trials. Therefore, our dataset contains the data from 12 pairs i.e. 24 participants. Each study session took approximately 15 minutes, and the participants were compensated with $5 for participation.

• **Data Processing and Annotations**

We used the KinectXEFTools library to extract the RGB videos from the raw Kinect recordings (“.xef” format). We used the Kinect2Mat library to extract the depth data in “.mat” format from the raw Kinect recordings. We split the data recordings of each study session into individual handovers manually. The data from the two Kinect sensors and the OptiTrack system was recorded on three separate computers. To help synchronize the three data streams during post-processing, we had asked participants to stand in a “T-pose” at the beginning of each study session. We used the timestamps of this event to synchronize the data recordings of the two Kinect sensors and the OptiTrack system. From the OptiTrack Motive software, we also exported the Quaternion orientations of 13 upper-body bones for each participant with respect to their parental segment, roughly representing the joint angles.

We annotated each frame of the data recordings with the three phases of a handover: reach, transfer, and retreat. The “reach” phase corresponds to the reaching motion of the giver and receiver before the receiver makes contact with the object. The “transfer” phase starts when both the giver and receiver touch the object for the first time and ends when the giver releases the object. In the “retreat” phase, the giver and receiver retract their hands to the resting position. In our annotations, the “transfer” phase corresponds to the portion of the handover where the distance between the giver's and receiver's hands is within 20cm of the minimum distance of the handover, and their relative velocity is less than 15cm/s. These thresholds were obtained with trial-and-error.

## Ethics Statements

The study protocol was approved by Cornell University's Institutional Review Board for Human Participants (Protocol ID#: 2111010686, Approval Date: January 24, 2022). Informed consent was obtained from the participants before the study.

## CRediT authorship contribution statement

**Alap Kshirsagar:** Conceptualization, Methodology, Software, Investigation, Data curation, Writing – original draft, Visualization. **Raphael Fortuna:** Software, Validation, Data curation, Writing – review & editing. **Zhiming Xie:** Data curation, Validation, Visualization, Writing – review & editing. **Guy Hoffman:** Conceptualization, Supervision, Writing – review & editing.

## Declaration of Competing Interests

The authors declare that they have no known competing financial interests or personal relationships that could have appeared to influence the work reported in this paper.

## Data Availability

Dataset of Bimanual Human-to-Human Object Handovers (Original data) (Zenodo).
